# Joint Convention of the American and Southern Dental Associations

**Published:** 1898-01

**Authors:** 


### JOINT CONVENTION OF THE AMERICAN AND SOUTHERN DENTAL ASSOCIATIONS.

Joint Convention, held in the pavilion of the Hygeia Hotel, Old Point Comfort, Va., on Thursday, August 5, 1897, at half-past twelve o'clock.

The meeting was called to order by Dr. Fillebrown.

Dr. John B. Rich, of Washington, was nominated Chairman of the meeting, and Dr. Walker, of Atlanta, and Dr. George H. Cushing, of Chicago, Temporary Secretaries.

*Dr. Patterson.*—I introduce the following resolution and move its adoption:

*Resolved*, That the members of the American Dental Association and the members of the Southern Dental Association do hereby organize themselves into a body to be known and styled as the National Dental Association.

Motion carried.

*Dr. Marshall.*—I would offer the following resolution:

*Resolved*, That the constitution, by-laws, and rules of order decided upon and presented by the joint committee of these two bodies be and are hereby adopted as the constitution, by-laws, and rules of order of the National Dental Association.

Resolution adopted.

*Dr. Richards.*—I desire to offer the following resolution:

*Resolved*, That no speaker shall occupy the floor for more than three minutes, without the consent of a majority of this body, during this session.

Resolution adopted.

*Dr. Ottofy.*—I move that we proceed to the election of officers.

Motion seconded.

*Dr. Ottofy.*—As there seems to be some objection to that at the present time, I move that the motion as to the election of officers be laid on the table.

Motion carried.

*Dr. Beadles.*—I move that the constitution and by-laws as adopted by this body be read by the Secretary.

Motion carried, and the Secretary read the same.

*The Chairman.*—You have heard the reading of this constitution, by-laws, and rules of order; they are now before you for action. What is your pleasure in regard to them?

*Dr. Marshall.*—The constitution, by-laws and rules of order have already been adopted; the gentleman merely asked to have them read.

*Dr. Ottolengui.*—I move that the motion to proceed with the election of officers be now taken from the table and acted upon.

Motion carried.

The Chairman appointed Dr. Noble and Dr. Hunt as tellers, and the election resulted as follows: President, Dr. Thomas Fillebrown, of Boston; Secretary, Dr. George H. Cushing, of Chicago; Assistant Secretary, Dr. W. E. Walker, of Atlanta; Vice-President from the East, Dr. James McManus, of Hartford, Conn.; Vice-President from the West, Dr. L. L. Dunbar, of San Francisco; Vice-President from the South, Dr. B. Holly Smith, of Baltimore; Corresponding Secretary, Dr. Emma Eames Chase, of St. Louis; Treasurer, Dr. Henry W. Morgan, of Nashville.

*Executive Committee.*[1]—Dr. G. L. Noel, Dr. J. N. Crouse, Dr. V. H. Jackson, for three years; Dr. M. F. Finlay, Dr. J. D. Patterson, Dr. H. A. Smith, for two years; Dr. George Eubank, Dr. W. P. Dickinson, Dr. C. N. Peirce, for one year.

Dr. Marshall then offered the following resolution:

*Resolved*, That the Treasurer of this Association be directed to request the Treasurers of the American Dental Association and the Southern Dental

---

[1] Appointed by the Chair, upon motion of Dr. Beadles.

Association to furnish him with a list of the permanent members in their respective Associations, and that he is hereby directed to add the names so furnished to the constitution of this Association, as set forth in the provision of membership, Article 3, Section 5, of the Constitution ; and be it further

*Resolved*, That such act of the Treasurer shall be legal and binding, the same as if the names had been attached by the persons themselves.

The place of meeting next year was then voted for; Dr. Crouse placed the cities of Omaha, Minneapolis, and Denver in nomination, Omaha being finally selected.

Dr. Marshall offered the following resolution :

*Resolved*, That a committee of three be appointed by the President of this Association to take into consideration the question of establishing a journal for the publication of the transactions of this Association and other original matter pertaining to dental surgery and the collateral sciences, and that they report to this body at the next annual meeting.

Resolution adopted.

Dr. Crawford offered the following resolution :

*Resolved*, That it is the sense of the National Dental Association that when any member of the dental profession in good standing presents a certificate of registration from any State in the Union he shall be admitted to registration in any other State of the Union when presenting such certificate of registration and good standing professionally, without an additional examination.

*Dr. Crawford.*—The moral force that that resolution carries with it will do more to deter young men in this country from engaging in the practice of dentistry illegally than anything else. Whether this Association endorses this sentiment or not, it is right. It is only advisory,—it is not compulsory,—but it carries with it all the force that moral suasion is capable of carrying with it.

*Dr. Donnelly.*—Who is to be influenced by it? Certainly not the boards that are governed by the laws of their own States. Is it directed to the Legislature or the examining boards?

*Dr. Crawford.*—To the examining boards.

*Dr. Marshall.*—It has always seemed to me that the laws of the States regulating the practice of dentistry, which deprived a citizen of one State from gaining his livelihood in another, would be declared unconstitutional if carried into the United States Supreme Court.

Dr. Crawford's resolution adopted.

*Dr. Crouse.*—We have adopted a constitution and by-laws; the printed copies that have been sent out have been corrected. This

Association should authorize Dr. Fillebrown to have corrected and printed a sufficient number of copies of the same.

Dr. Morgan offered the following resolution:

*Resolved*, That the two Associations, the American Dental Association and the Southern Dental Association, participating in this organization be requested to contribute one dollar per member to the treasury of the National Dental Association, and that in view of this contribution all dues be remitted from such names.

Resolution adopted.

Dr. Hunt offered the following resolution:

*Resolved*, That a committee of three be appointed by the President of this Association to report at the next annual meeting measures looking to the preparation of the full history of the dental profession.

Resolution adopted.

